# Prevalence of Parent-Reported Food Allergies Among Children in Saudi Arabia

**DOI:** 10.3390/nu16162693

**Published:** 2024-08-14

**Authors:** Ibrahim Alibrahim, Maria AlSulami, Turki Alotaibi, Ruba Alotaibi, Elaf Bahareth, Inam Abulreish, Sumayyah Alsuruji, Imad Khojah, Loie Goronfolah, Husni Rayes, Ameera Bukhari, Amer Khojah

**Affiliations:** 1 College of Medicine, Umm Al-Qura University, Makkah 21955, Saudi Arabia; i.s.alibrahim0@gmail.com (I.A.); mariaaalsulami@gmail.com (M.A.); turkialotaibi320@gmail.com (T.A.); rubazaid044@gmail.com (R.A.); el-elaf@hotmail.com (E.B.); en3amar@hotmail.com (I.A.); medicalst.42@gmail.com (S.A.); 2 Faculty of Medicine, King Abdulaziz University, Jeddah 21589, Saudi Arabia; ikhojah@kau.edu.sa; 3 King Abdullah International Medical Research Center, King Saud Bin Abdulaziz University for Health Sciences, Jeddah 22384, Saudi Arabia; loie6@hotmail.com; 4 Department of Pediatrics, Makkah Maternity and Children Hospital, Makkah 24246, Saudi Arabia; drhusni2002@yahoo.com; 5 College of Science, Taif University, Taif 21944, Saudi Arabia; a.bukhari@tu.edu.sa; 6 Department of Pediatrics, College of Medicine, Umm Al-Qura University, Makkah 21955, Saudi Arabia

**Keywords:** food allergy, atopy, pediatric allergy, Saudi Arabia

## Abstract

(1) Background: Food allergy (FA) is an immune-mediated hypersensitivity to foods, significantly contributing to childhood morbidity and mortality. This study aimed to assess the prevalence, characteristics, and influencing factors of parent-reported FAs among children in Saudi Arabia. (2) Methods: This cross-sectional study utilized a validated parental questionnaire distributed across all regions of Saudi Arabia. Data from 2130 participants were collected and analyzed using SPSS v. 26 and Prism software v. 10.3.0. (3) Results: Parent-reported FA prevalence was 15.2%. Egg was the most common allergen (6.2%), followed by tree nuts (4.1%), peanuts (4.0%), milk (3.8%), and sesame (3.2%). Significant geographical variations were observed, with the western region having the highest burden (*p* < 0.001). Older children had higher rates of shellfish and fish allergies. Parental allergies and co-existing asthma/drug allergies were positively associated with childhood FAs. (4) Conclusions: This study highlights a substantial burden of parent-reported FAs in Saudi Arabia, with regional variations in food allergen distribution. Parental allergies and co-existing allergic conditions may influence FA risk.

## 1. Introduction

Food allergy (FA) is a hypersensitivity (immune-mediated) reaction against certain types of food that are generally considered safe, but which the immune system mistakenly perceives as harmful [1]. FA is a significant contributor to childhood morbidity, responsible for numerous cases of anaphylaxis and other severe allergic reactions [2,3]. While FA can affect all age groups, studies consistently demonstrate a higher prevalence in young children, potentially impacting their quality of life [4]. Various factors, including genetics, environment, and dietary habits, are believed to contribute to FA development [5,6]. FA manifestations range from mild skin symptoms (pruritus, urticaria, and angioedema) to life-threatening anaphylaxis requiring immediate medical intervention [7]. Fortunately, injectable epinephrine administration is the first-line and most effective treatment of food-induced anaphylaxis, reversing the reaction and potentially saving the patient’s life [8]. However, delayed administration is associated with increased mortality rates [9].

Several studies have investigated the prevalence and characteristics of food allergies (FAs) in children across different geographical regions. For example, Alzahrani et al. conducted a cross-sectional study in Taif City and reported a parent-reported FA prevalence of 16.1%, with eggs, peanuts, and sesame as the most common culprits [10]. In contrast, Al Ghadeer et al. found a higher reported prevalence of FAs at 34.4% of study subjects, in the eastern region of Saudi Arabia, with fish as the most common trigger [11]. Al-Hammadi et al. in Al-Ain City, the United Arab Emirates, documented a lower prevalence (8%) of physician-diagnosed FAs in children, with eggs, fruits, and fish as the most common triggers [12]. These variations emphasize the need for a comprehensive, nationwide study in Saudi Arabia to examine the regional variation in the rate of parent-reported FAs and identify the most prevalent allergens within each geographical region. This study aimed to assess the prevalence, characteristics, and influencing factors of parent-reported FAs among children in Saudi Arabia, highlighting the burden of FAs in the country and regional variations in food allergen distribution.

## 2. Materials and Methods

This cross-sectional study was conducted between August 2023 and February 2024 in the Kingdom of Saudi Arabia. The data were collected via the use of a validated self-administered questionnaire obtained from previously published studies [10,13]. It was distributed through text messages and various social media platforms (X, WhatsApp, Instagram, and Facebook), with 42 data collectors in the western, eastern, central, southern, and northern regions to ensure comprehensive coverage of all geographical areas within the Kingdom of Saudi Arabia. The questionnaire was translated into Arabic, the native language of the target population, and built using Google Forms (https://www.google.com/intl/en-GB/forms/about/#overview, accessed on 24 June 2024). It was validated through pilot testing with around 20 participants and was pre-tested to ensure its validity. The target population was all parents of children younger than 18 years and resident in Saudi Arabia. Exclusion criteria included individuals employed in the medical field, those declining participation, and those who failed to complete the questionnaire.

The sample size calculation utilized Epi info software version 2.1 [14]. Assuming a population size of 32.175.224 individuals (based on the 2022 Saudi census) [15], a 95% confidence interval (CI), and a 50% anticipated response rate, a minimum sample size of 385 participants was determined. However, the final sample size comprised 2130 participating parents.

The survey instrument consisted of three sections. The first section gathered demographic information regarding the child’s age, gender, and nationality, along with parental age and socioeconomic status. The second section included questions about the parents’ allergy history and the child’s FAs. Finally, the third section addressed parents’ attitudes and practices regarding the management of their children’s FAs. Informed consent was obtained from all participants prior to survey participation.

Ethical approval for this study was obtained from the UQU institutional research board (Approval No. HAPO-02-K-012-2023-09-1759, date 28 September 2023). The link had the survey’s objectives and a request to participate in a voluntary questionnaire. The data were inputted into Microsoft Excel and analyzed using version 26 of SPSS software and Prism software version 10.3.0. Associations between outcome and predictor variables were analyzed using chi-square, and the level of statistical significance was set at *p* < 0.05.

## 3. Results

### 3.1. Demographic Characteristics of the Study Participants

A total of 2130 participants were included in the study. Gender distribution revealed a higher proportion of female parents (67.9%) compared to males (32.1%). Geographical representation across regions revealed a slight predominance in the western region (23.8%), followed closely by the southern and central regions (20.5% and 20.1%) (Table 1). The gender distribution of children slightly favored males (52.5%) over females (47.5%). Data regarding parental allergies showed varying prevalence rates, with around 60% of parents reporting no allergic diseases. Among those affected with allergic diseases, asthma and allergic rhinitis were the most prevalent conditions (Table 1). Additional demographic information is demonstrated in Table 1.

**Figure 1:**
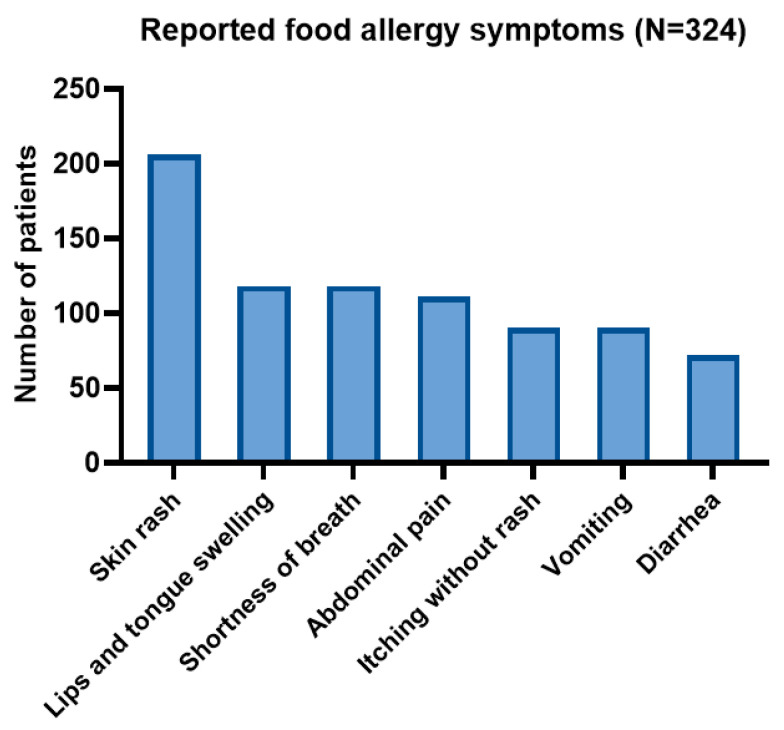
Symptoms frequency in children with reported food allergy.

There were no significant demographic differences between children with and without parent-reported FAs, except for the place of residence (*p* < 0.001) (Table 2). Most children with a food allergy presented with cutaneous symptoms, followed by gastrointestinal and then respiratory symptoms. The frequency of these symptoms is presented in Figure 1.

### 3.2. Prevalence and Regional Variations in Parent-Reported FAs among Saudi Arabian Children

**Figure 2:**
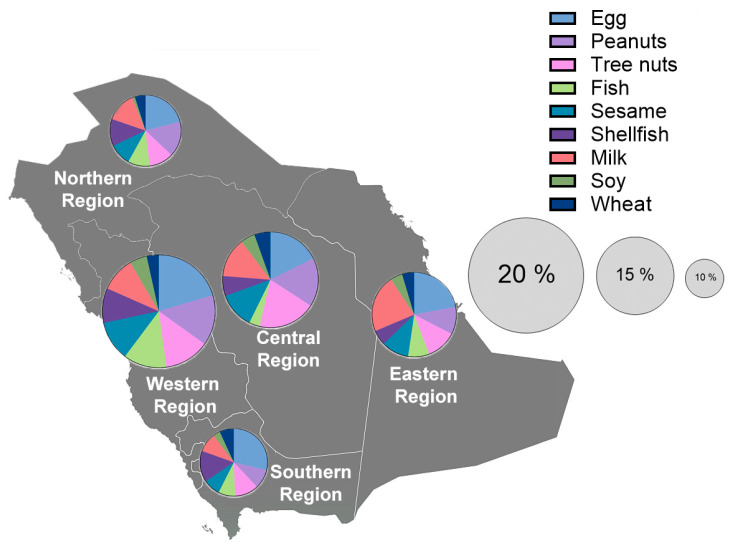
Prevalence of parent-reported food allergies among children in different regions in Saudi Arabia. The size of the pie chart reflects the prevalence and the color reflects the frequency of the 9 major food allergens.

The prevalence of parent-reported FAs was 15.2%. This prevalence, however, displayed significant geographical heterogeneity (Chi2 *p* < 0.001; Table 2). The western region exhibited the highest burden of FAs (20.5%), followed by the central (16.6%), eastern (13.6%), northern (12.3%), and southern regions (11.7%) (Figure 2). Egg (6.2%) was the most commonly reported food allergen, followed by tree nuts (4.1%), peanuts (4%), milk (3.8%), and sesame (3.2%) (Table 3). However, there were some regional variations. For example, tree nuts were the most common allergen in the central region. While shellfish was the sixth most common allergen in the overall study, it was the second most common trigger in the southern region. Similarly, milk was the second most common trigger in the eastern region. Lastly, fish was the least common allergen out of the nine major allergens in the central region, while it was the third most common in the western region (Figure 2, Supplemental Figure S1).

### 3.3. The Association between Age and Parent-Reported FAs

**Figure 3:**
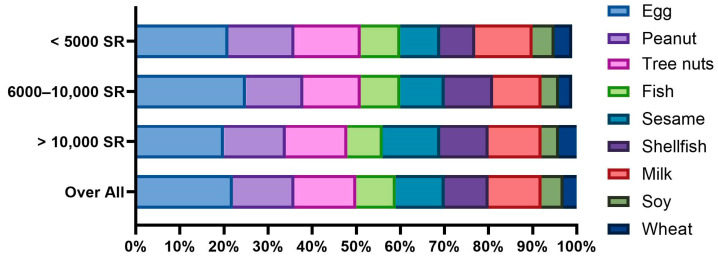
Prevalence of parent-reported food allergies by age group.

We divided the study participants into the following three age groups: ≤5 years (29.2%), 6–10 years (36.2%), and 11–18 years (34.6%). The prevalence of FAs did not differ significantly between groups (14.4%, 14.8%, and 16.2%, respectively; chi-squared test, *p* = 0.615; Table 2). However, the distribution of specific food allergens varied with age. Shellfish and fish allergies appeared to be more prevalent in older age groups, while milk and soy allergies seemed to decrease with age (Figure 3).

**Figure 4:**
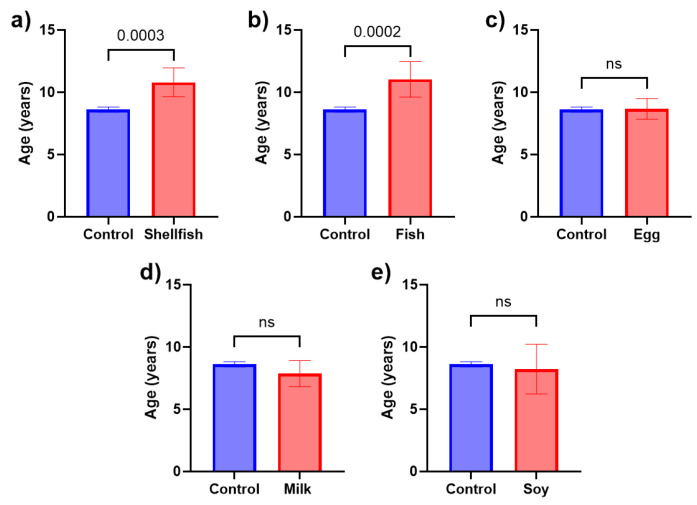
The mean age of children with food allergy vs. controls. (**a**) The average age of shellfish-allergic patients was higher than controls (10.8 ± 4.4 years vs. 8.6 ± 4.5 years; *p* = 0.0003). (**b**) Patients with fish allergy were older (11 ± 5 years) compared to controls (8.6 ± 4.5 years; *p* = 0.0002). (**c**–**e**) The average age of egg-, milk-, and soy-allergic individuals did not differ significantly from controls. “ns” means not significant.

To further investigate this observation, we compared the mean age of control subjects (children without FAs) with patients with specific food allergies. The results demonstrated a statistically significant difference in mean age for both fish and shellfish allergies. Patients with a fish allergy were older (11 ± 5 years) compared to controls (8.6 ± 4.5 years; *p* = 0.0002). Similarly, the average age of shellfish-allergic patients was higher (10.8 ± 4.4 years; *p* = 0.0003). Conversely, the mean age of milk- and soy-allergic individuals (7.9 ± 4.5 years and 8.2 ± 5.0 years, respectively) was lower compared to controls, although these differences were not statistically significant (Figure 4).

### 3.4. Parental Allergies and Comorbidities in Children with FAs

Among 324 children with FAs, 54.4% had fathers with allergies, significantly higher than the 35.9% of fathers without allergies (*p* < 0.001). Similarly, 55.9% of these children had mothers with allergies compared to 35.5% without maternal allergies (*p* < 0.001). In contrast, among 1795 children without FAs, the percentages of fathers and mothers with allergies were notably lower (Table 4).

Children with FAs also demonstrated a higher prevalence of coexisting asthma (*p* < 0.001) and drug allergies (*p* = 0.007) compared to controls. However, no significant difference was observed in the prevalence of rhinitis (Table 4).

### 3.5. Characteristics of Children with Single and Multiple Food Allergies

**Figure 5:**
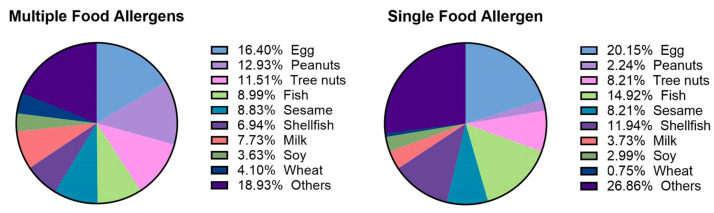
The distribution of the food allergens in children with single and multiple food allergies.

Out of the 324 participants, 132 children had a single food trigger for their allergy, while 192 had multiple triggers. The distribution of the triggers varied between these groups, with egg, fish, and shellfish being predominant in the single-allergen group, whereas egg, peanuts, and tree nuts were more common in the multiple-allergen group (Figure 5). A history of allergies in both fathers and mothers was significantly associated with the reporting of multiple food allergies in their children (*p* = 0.028 and *p* = 0.027, respectively).

## 4. Discussion

Food allergy is considered the most common cause of anaphylaxis in children and is a significant contributor to pediatric morbidity and mortality [2,16,17]. This study aimed to determine the parent-reported prevalence of FAs among Saudi Arabian children and identify the most common food triggers. The prevalence rate of FAs among our population was 15.2%, which is consistent with a previously reported prevalence of 16.1% in TaifCity, Saudi Arabia [10]. Conversely, a study conducted in the Eastern Province of Saudi Arabia exhibited a higher prevalence rate of 34.4% [11]. Studies from the Gulf region revealed lower prevalence rates, with 4.1% among children aged 11 to 14 in Kuwait and 8% among school children in the Emirates [12,18]. A study from Jordan reported a parent-reported FA prevalence of 11.2%, although only one-third of these cases were confirmed by physicians [19]. These discrepancies suggest potential overestimation by parents potentially mistaking food intolerance for FAs. To mitigate this, our study excluded participants reporting non-allergic symptoms (constipation and hemolysis) and investigated if the diagnosis was made by a physician. We found that 39% of diagnoses were made by allergists, 19.3% by general pediatricians, and 41.7% were self-diagnosed. These results are in line with reports from the United States, which found that 30% of parent-reported food allergies were self-diagnosed [20].

International studies on parent-reported FAs yielded variable results as follows: 6.2% in China, 11.7% in Brazil, and 7.6% in the United States [21,22,23]. Interestingly, we observed significant geographical variations in prevalence. The western and central regions, encompassing larger urban centers in Saudi Arabia, had a higher prevalence compared to the more rural and agricultural northern and southern regions. This aligns with a large US population study demonstrating a higher prevalence (9.8%) in urban children compared to rural ones (6.2%) [24,25]. Additionally, a German study reported lower FA rates in farmers (3.3%) compared to indoor workers (7.0%) [26]. These findings suggest potential environmental influences on FA development.

Regarding food allergens, eggs emerged as the most common reported food allergen in most regions of Saudi Arabia (6.2%). This result is similar to previously published studies in Taif, Saudi Arabia, and the Emirates [10,12,27]. In contrast, studies from China, Korea, and Brazil identified seafood as the most prevalent allergen [21,22,28,29], while peanuts were the most common allergen in the United States [23,30]. These variations likely reflect dietary differences across countries, including food consumption patterns, preparation methods, and age of introduction [31]. Interestingly, our study revealed regional variations within Saudi Arabia. Fish allergy was relatively uncommon in the central region, which is geographically distant from major bodies of water. This finding supports the notion that dietary patterns can influence the prevalence of specific food allergies.

The effect of age on the prevalence of food allergy remains a topic of ongoing investigation, with conflicting results. While some studies suggest a diminishing prevalence of food allergy with age, others report a potential increase [5,32,33]. However, the majority of the existing literature identifies milk as the predominant food allergen in early childhood (less than 2 years old), demonstrating a significant decline in prevalence over time [34,35,36]. We did not observe a significant change in overall food allergy prevalence over time within our study population. However, our observations were consistent with other studies concluding that milk allergies seem to decrease over time, while fish and shellfish allergies appear more prevalent in older patients [37]. This observation aligns with data from the United States, where shellfish allergies emerge as the most common adult food allergy [38,39], contrasting with peanut and milk allergies in pediatric populations [23,40]. This trend is expected, given that most individuals outgrow milk and egg allergies, while shellfish and fish allergies are more likely to persist into adulthood [41,42,43,44,45].

Regarding allergic atopy comorbidities, we found a significant association between a history of drug allergy and asthma with parent-reported food allergy. This is consistent with other studies that have shown a positive association between food allergy in pediatric patients and other atopic disorders [46,47,48,49,50]. We also found a positive association between a paternal history of atopy and reported pediatric food allergy. This is consistent with a large cohort study from Japan and Australia [51,52]. Lastly, we observed a high prevalence of multiple food allergies (around 60% of children with reported food allergy) and found a positive association between family history and reporting multiple food allergies. The rate of multiple food allergies was higher than that of a large-scale study from the United States, which showed a rate of around 30% [30]. This discrepancy could be due in part to the higher prevalence of family history of atopy or the rising rate of food allergies in general [53,54,55,56]. Multiple food allergies are a major concern and can limit the diets of children, leading to nutritional deficiency [57,58]. Fortunately, there are new therapeutic approaches to treat these patients, such as omalizumab, a monoclonal anti-IgE antibody, which has been in trial for patients with multiple food allergies, and eosinophilic esophagitis [59,60]. Additionally, there have been many successful trials of food desensitization [61,62].

Despite the high prevalence of reported food allergies in our study, there seems to be a knowledge gap in the management of food allergy among the public and schoolteachers in Saudi Arabia [63,64,65]. We hope this study will raise awareness of the magnitude of the problem and encourage policymakers to allocate more resources towards raising awareness and promoting strategies aimed at lowering the prevalence of food allergies, such as the early introduction of allergenic foods.

One of the strengths of this study is the good sample size and inclusion of all the different regions in the country, allowing us to examine regional differences and risk factors for food allergies, such as a history of atopy and family history.

Study limitations include the reliance on parent reports, which may lead to overestimating the true prevalence of food allergies. Additionally, this study did not use the gold-standard test for food allergies, which is the double-blind placebo-controlled (DBPC) food challenge test, due to logistical infeasibility and the possible selection bias that would occur. The DBPC food challenge test is currently performed in large food allergy centers and many parents refuse testing due to the risk of severe reactions. Furthermore, this study did not explore the barriers to seeking physician diagnosis for 40% of the patients or whether they are receiving optimal care, including the availability of injectable epinephrine and immunological testing. Future studies may explore the utilization rate of injectable epinephrine and its impact on care, as well as the impact of food allergies on patients’ quality of life. It would also be interesting to repeat this study in the future after the implementation of new guidelines that recommend early food introductions to examine the impact of this intervention on the rate of parent-reported food allergies.

## 5. Conclusions

This study highlights a substantial burden of parent-reported FAs in Saudi Arabia, with regional variations in food allergen distribution. Parental allergies and co-existing allergic conditions may influence FA risk.

## Figures and Tables

**Table 1:** Sociodemographic characteristics of study participants (N = 2130).

Characteristic		Frequency	Percentage
Parent’s gender	Female	1447	(67.9%)
Parent’s age	Less than 26 years	224	(10.5%)
	27–35 years	505	(23.7%)
	36–45 years	785	(36.9%)
	46–55 years	602	(28.3%)
	More than 55 years	14	(0.7%)
Region	Western region	507	(23.8%)
	Eastern region	375	(17.6%)
	Central region	429	(20.1%)
	Southern region	437	(20.5%)
	Northern region	382	(17.9%)
Educational level	High school level or lower	417	(19.6%)
	Bachelor’s degree	1490	(70.0%)
	Master’s, PhD, or equivalent	223	(10.5%)
Family monthly income	Less than SR 1000	237	(11.1%)
	SR 1000–SR 5000	415	(19.5%)
	SR 6000–SR 10,000	563	(26.4%)
	More than SR 10,000	915	(43.0%)
Gender of the child	Female	1011	(47.5%)
Child’s age	5 years and below	131	(6.2%)
	6 years–10 years	534	(25.1%)
	11 years–18 years	578	(27.1%)
Father’s allergies ^1^	Asthma	292	(13.7%)
	Allergic rhinitis	471	(22.1%)
	Food allergy	163	(7.7%)
	Drug allergy	57	(2.7%)
	None	1304	(61.2%)
Mother’s allergies ^1^	Asthma	239	(11.2%)
	Allergic rhinitis	504	(23.7%)
	Food allergy	197	(9.2%)
	Drug allergy	102	(4.8%)
	None	1307	(61.4%)

^1^ Some participants had more than one allergic condition.

**Table 2:** The association between children with food allergy and sociodemographic factors.

Characteristic		Children without FA (N = 1805)	Children with FA (N = 324)	*p*-Value
Parent’s gender	Male	1219 (67.5%)	228 (70.4%)	0.308
	Female	587 (32.5%)	96 (29.6%)	
Parent’s age	Less than 26 years	199 (11%)	25 (7.7%)	0.184
	27–35 years	422 (23.4%)	83 (25.6%)	
	36–45 years	659 (36.5%)	126 (38.9%)	
	46–55 years	516 (28.6%)	86 (26.5%)	
	More than 55 years	10 (0.5%)	4 (1.2%)	
Region	Western region	386 (21.4%)	51 (15.7%)	<0.001
	Eastern region	324 (17.9%)	51 (15.7%)	
	Central region	335 (15.6%)	47 (14.5%)	
	Southern region	403 (22.3%)	104 (32.1%)	
	Northern region	358 (19.8%)	71 (21.9%)	
Educational level	High school or lower	359 (19.8%)	58 (17.9%)	0.175
	Bachelor’s degree	1267 (70.2%)	223 (68.8%)	
	Master’s, PhD, or equivalent	180 (10%)	43 (13.3%)	
Family monthly income	Less than SR 1000	202 (11.2%)	35 (10.8%)	0.284
	SR 1000–SR 5000	348 (19.3%)	67 (20.7%)	
	SR 6000–SR 10,000	466 (25.8%)	97 (29.9%)	
	More than SR 10,000	790 (43.8%)	125 (38.6%)	
Gender of the child	Male	867 (48%)	144 (44.4%)	0.237
	Female	939 (52%)	180 (55.6%)	
Child’s age	5 years and below	517 (28.6%)	87 (26.9%)	0.615
	6 years–10 years	639 (35.4%)	111 (34.3%)	
	11 years–18 years	599 (33.2%)	116 (35.8%)	

**Table 3:** Prevalence of parent-reported food allergy and the 9 major food allergens.

	Frequency	Percentage
Any food allergy	324	15.2%
Egg	131	6.15%
Tree nuts	87	4.08%
Peanuts	85	4.0%
Milk and dairy products	80	3.76%
Sesame	67	3.15%
Shellfish	59	2.77%
Fish	53	2.49%
Wheat	30	1.41%
Soybean	26	1.22%
Other allergens ^1^	160	7.5%

^1^ “Other allergens” encompasses a wider range of food triggers, detailed in Supplemental Table S1.

**Table 4:** Parental allergies and comorbidities in children with FAs.

Characteristic		Children with FA (N = 324)	Children without FA (N = 1805)	*p*-Value
Father’s Allergies ^1^	Present	177 (21.4%)	649 (78.6%)	<0.001
	Absent	147 (11.3%)	1157 (88.7%)	
Mother’s Allergies ^1^	Present	182 (22.1%)	641 (77.9%)	<0.001
	Absent	142 (10.9%)	1165 (89.1%)	
Drug Allergies (Child)	Present	13 (29.5%)	31 (70.5%)	0.007
	Absent	311 (14.9%)	1775 (85.1%)	
Asthma (Child)	Present	89 (25.4%)	261 (74.6%)	<0.001
	Absent	235 (13.2%)	1545 (86.8%)	
Rhinitis (Child)	Present	43 (16.6%)	216 (83.4%)	0.506
	Absent	281 (15.0%)	1590 (85.0%)	

^1^ “Allergies” refers to the presence of any of the following conditions: asthma, allergic rhinitis, food allergy, and drug allergy.

## Data Availability

The raw data supporting the conclusions of this article will be made available by the authors upon reasonable request.

## Supplementary Materials

The following supporting information can be downloaded at: https://www.mdpi.com/article/10.3390/nu16162693/s1, Figure S1: Prevalence of parent-reported food allergy by geographical region; Table S1: Prevalence of other reported food allergens.
