# Supplementary material for: Prevalence of Parent-Reported Food Allergies Among Children in Saudi Arabia
Source: Nutrients. 2024 Aug 14;16(16):2693. doi: 10.3390/nu16162693 (PMC11356889; doi:10.3390/nu16162693)
Supplement: Supplementary file 1 [file nutrients-16-02693-s001.zip › nutrients-3109327-supplementary.pdf]

Table S1. Prevalence of reported food allergens “others”.

	Frequency	Percentage
Banana	61	2.87%
Mango	48	2.25%
Kiwi	44	2.07%
Strawberry	37	1.74%
Eggplants	26	1.22%
Blueberry	17	0.80%
Date	10	0.47%
Chocolate	8	0.38%
Meat	4	0.19%
Honey	2	0.1%
Lentil	2	0.1%
Coffee	1	0.05%
Other fruits*	17	0.80%
Other vegetables**	9	0.42%

* Other fruits that the participants mentioned are Fig, Orange, Peach, Pineapple, Cantaloupe, Grape, Pomegranate, and Watermelon; ** Other vegetables that the participants mentioned are Tomato, Bell Pepper, Broccoli, Chili Pepper, Corn, and Potato; *** Others include Date, Chocolate, Meat, Honey, Lentil, Coffee, and Rice.

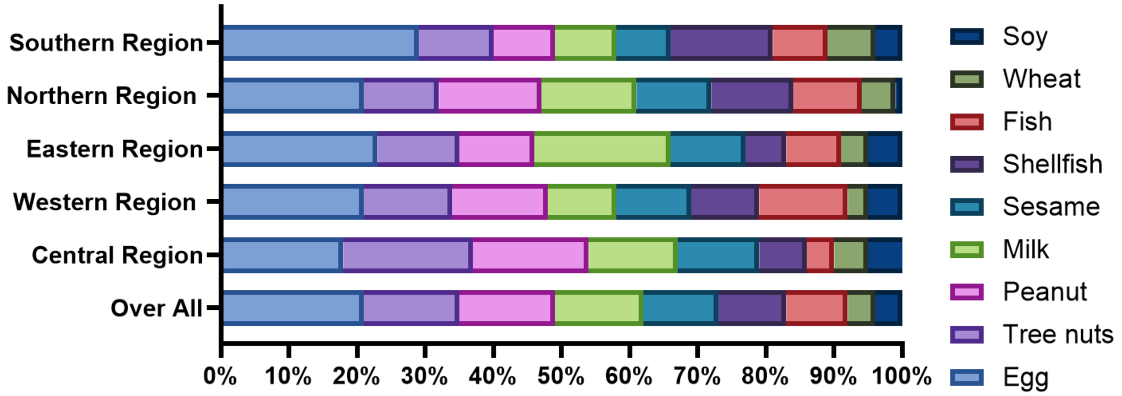

Supplemental Figure S1. Prevalence of parent-reported food allergy by geographical region.
